# Prevalence, potential virulence genes, and antimicrobial resistance of *Aeromonas* spp. in farm-raised *Oreochromis niloticus* and *Labeo rohita* in Noakhali, Bangladesh

**DOI:** 10.1371/journal.pone.0347577

**Published:** 2026-04-28

**Authors:** Noimul Hasan Siddiquee, Imam Hossain, Popy Devnath, Farzana Islam, Rahima Akter, Mridul Gope Topu, Israt Jahan Mitu, Md Abdul Hannan, Md. Shariful Islam, Mohammad Sharif Uddin, Sutapa Bhowmik

**Affiliations:** 1 Department of Microbiology, Noakhali Science and Technology University, Noakhali, Bangladesh; 2 Bioinformatics Laboratory (BioLab), Noakhali, Bangladesh; Tanta University Faculty of Agriculture, EGYPT

## Abstract

**Background:**

*Aeromonas* species are ubiquitous aquatic bacteria and opportunistic pathogens associated with motile *Aeromonas* septicemia (MAS) in freshwater fish. MAS is characterized by hemorrhagic lesions and septicemia and can cause severe economic losses in aquaculture. The increasing occurrence of antimicrobial-resistant *Aeromonas* strains has raised concerns for fish health, aquaculture sustainability, and public health.

**Aim:**

This study aimed to determine the prevalence, species distribution, virulence gene profiles, and antimicrobial resistance patterns of *Aeromonas* spp. isolated from farm-raised *Oreochromis niloticus* and *Labeo rohita* collected from local markets in Noakhali, Bangladesh.

**Methods:**

A total of 22 *Aeromonas* isolates were obtained from intestinal samples and characterized using biochemical assays and PCR amplification of the *gyrB* gene. Species-level identification and phylogenetic relationships were determined by 16S rRNA gene sequencing. Antimicrobial susceptibility was evaluated using the disc diffusion method following CLSI guidelines, and multiple antibiotic resistance (MAR) was calculated for each isolate. PCR-based screening of nine virulence-associated genes was performed to assess pathogenic potential.

**Results:**

Phylogenetic analysis identified five *Aeromonas* species, with *A. veronii* as the predominant species. Several virulence genes, particularly *act*, *alt*, and *ast*, were frequently detected among the isolates. High levels of antimicrobial resistance were observed against β-lactam antibiotics, and MAR index analysis indicated that many isolates were multidrug resistant.

**Conclusion:**

These findings highlight the presence of virulence gene and multidrug-resistant *Aeromonas* in farm-raised fish in Bangladesh. Continuous surveillance and responsible antimicrobial use are essential to mitigate potential risks to aquaculture and public health.

The graphical abstract of this study is illustrated in Fig 1.

**Fig 1:**
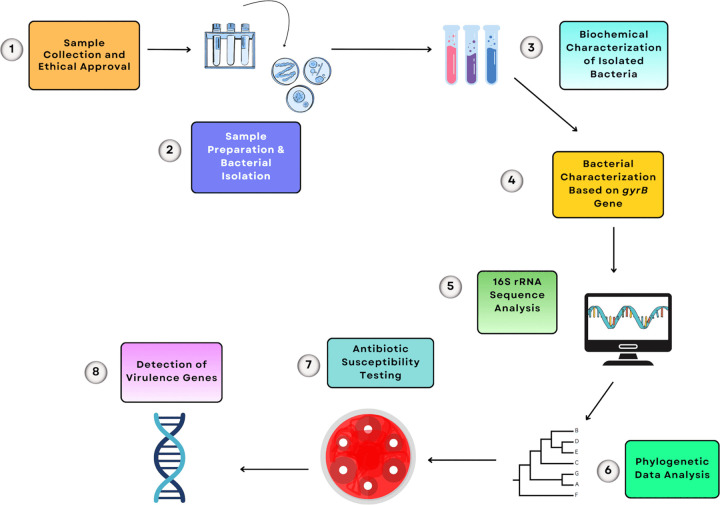
Workflow showing bacterial isolation, biochemical and molecular identification, phylogenetic analysis, antibiotic susceptibility testing, and virulence gene detection.

## 1. Introduction

Aquaculture has expanded rapidly worldwide in recent decades, driven by the depletion of wild seafood stocks and increasing demand for sustainable animal protein, making it indispensable for economic development and food security [1,2]. Bangladesh is the fourth-largest inland fish producer globally after China, India, and Myanmar. The country’s aquaculture sector continues to expand rapidly, with an annual production growth rate of approximately 8%, and supports the livelihoods of over 17 million people [3–5]. *Labeo rohita* (Rohu) and *Oreochromis niloticus* (Tilapia) are the most widely consumed fish species in Bangladesh, accounting for 25% and 8.95% of total production, respectively [4,6]. Despite these socioeconomic benefits, the aquaculture industry in Bangladesh faces persistent threats from infectious diseases caused by a diverse array of pathogens [7]. These pathogens include bacteria (e.g., *Aeromonas* spp., *Vibrio* spp.), viruses (e.g., iridoviruses and herpesviruses), fungi and oomycetes (e.g., *Saprolegnia* spp.), and parasites [8–12]. Among these, bacterial infections, particularly those due to *Aeromonas* spp., are especially devastating, causing mass mortalities and severe economic losses in freshwater aquaculture systems [13,14]. Multiple *Aeromonas* species—including *A. hydrophila*, *A. salmonicida*, *A. caviae*, *A. sobria*, *A. veronii*, and *A. jandaei*—infect fish and humans, causing diseases such as Motile *Aeromonas* Septicemia (MAS) and Epizootic Ulcerative Syndrome (EUS) [15,16]. In fish, these diseases commonly cause loss of appetite, skin hemorrhages, exophthalmia, and abnormal swimming [17–21]. Intensive farming practices, poor water quality, overcrowding, and unhygienic handling exacerbate the transmission and severity of these diseases [1]. To mitigate infections, antibiotics such as β-lactams, quinolones, and sulfonamides are routinely administered [2]. However, the indiscriminate and excessive application of these drugs has contributed to the emergence of multidrug-resistant (MDR) *Aeromonas* spp., posing significant threats to aquatic ecosystems, aquaculture productivity, and public health through the dissemination of resistance genes [2–4,16,22,23]. Effective management requires ongoing surveillance of resistance patterns and virulence genes [24].

*Aeromonas* spp. are opportunistic pathogens in aquaculture and possess numerous virulence factors that contribute to disease development in fish and humans. These bacteria produce extracellular toxins and enzymes, including enterotoxins, hemolysins, proteases, and cytotoxins, which disrupt epithelial barriers and damage host tissues [25–27]. Infection typically occurs through the skin, gills, or gastrointestinal tract, leading to systemic disease such as hemorrhagic septicemia in fish [21]. Several virulence genes, including those encoding toxins (*act*, *ast*, *alt*), cytotoxins (*aerA*, *hlyA*), and adhesion or motility factors (*laf*), have been widely used as molecular markers to evaluate the pathogenic potential of *Aeromonas* isolates [28–32]. Therefore, monitoring virulence gene profiles alongside antimicrobial resistance is important for understanding the pathogenic risk of *Aeromonas* in aquaculture systems [33,34].

This study aimed to investigate the prevalence, species distribution, virulence gene profiles, and antimicrobial resistance patterns of *Aeromonas* spp. isolated from farm-raised *Oreochromis niloticus* and *Labeo rohita* collected from local markets in Noakhali, Bangladesh. Understanding these characteristics is essential for assessing the potential risks posed by *Aeromonas* to aquaculture sustainability and public health.

## 2. Materials & methods

### 2.1. Sample collection and ethical approval

**Fig 2:**
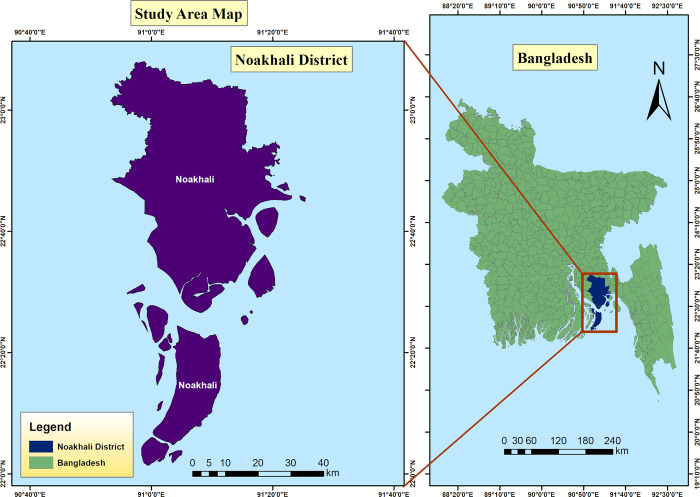
Geographical location of the sampling area in Bangladesh. The map was created using ArcGIS Pro (Esri, Redlands, CA, USA). Basemap data were obtained from Esri World Ocean Base (https://www.arcgis.com/home/item.html?id=6348e67824504fc9a62976434bf0d8d5), and administrative boundary layers were obtained from Esri World Administrative Divisions (https://www.arcgis.com/home/item.html?id=1b243539f4514b6ba35e7d995890db1d) via ArcGIS Online.

In this study, twenty-two random farm-raised healthy *O. niloticus* (n = 11) and *L. rohita* (n = 11) fish samples were collected from local vendors in the Noakhali region of Bangladesh (Fig 2 and Table 1). No signs or symptoms of infection were observed at the time of sampling, as only healthy appearing fish were available in the markets (Figure 1 in S1 File). The entire procedure was executed in compliance with the guidelines specified in the Guide for the Care and Use of Laboratory Animals by the National Institutes of Health, and ethical approval was also taken from the Noakhali Science and Technology University ethical committee with reference number NSTU/SCI/EC/2025/330.

**Table 1:** The weight (g) and Length (cm) of different samples collected for the study.

Sample Number	Sample Name	Weight (g)	Length (cm)
1	*L. rohita*	628	36
2	*O. niloticus*	168	21
3	*L. rohita*	725	38
4	*O. niloticus*	346	27
5	*O. niloticus*	220	24
6	*L. rohita*	656	40
7	*L. rohita*	611	38
8	*O. niloticus*	333	31
9	*L. rohita*	950	40
10	*O. niloticus*	460	31
11	*L. rohita*	470	34
12	*O. niloticus*	330	27
13	*O. niloticus*	275	25
14	*O. niloticus*	250	22
15	*O. niloticus*	365	25
16	*L. rohita*	1060	47
17	*O. niloticus*	500	29
18	*L. rohita*	400	33
19	*L. rohita*	600	35
20	*O. niloticus*	400	30
21	*L. rohita*	1000	46
22	*L. rohita*	865	39

### 2.2. Sample preparation & bacterial isolation

Fish surfaces were disinfected with 70% ethanol, and intestinal samples were aseptically collected [35] and samples were homogenized in sterile PBS [36]. Then, 100 μL of homogenized aliquots were plated on MacConkey agar plate and incubated at 37 °C for 24 h [37,38]. Although MacConkey agar is not a selective medium specifically designed for *Aeromonas* isolation, it allows preliminary differentiation of Gram-negative bacteria based on lactose fermentation. Therefore, suspected non-lactose-fermenting colonies were further confirmed using biochemical and molecular identification methods [39].

### 2.3. Biochemical characterization of isolated bacteria

Non-lactose-fermenting bacterial colonies on MacConkey’s agar plates were selected and purified. Then, isolated pure colonies were subjected to a series of biochemical tests, such as triple sugar iron (TSI), motility indole urea (MIU), hydrogen sulfide production (H_2_S), citrate, and oxidase [40], according to Bergey’s manual, 9^th^ Edition [41], to identify the suspected *Aeromonas.*

### 2.4. Bacterial characterization based on *gyrB* gene

PCR (Polymerase Chain Reaction) was used for molecular identification of all suspected *Aeromonas* isolates. All *Aeromonas* isolates identified by biochemical tests were cultivated overnight in Tryptic Soy Broth (TSB) at 37 °C for 24 h [40]. Subsequently, bacterial DNA was extracted from these cultures using the AddPrep bacterial genomic DNA extraction kit (addbio, Korea), following the manufacturer’s provided protocol. The isolated DNA was stored at −20 °C. A particular segment of the DNA *gyrase B* (*gyrB*) gene [42], with a PCR product size of around 1100 base pairs, was selected for the molecular identification of these isolates. The PCR reactions were conducted in the following manner: The final volume of each PCR reaction was 25 μL, comprising 12.5 μL of Promega 2X PCR mastermix, 0.6 μL of each forward and reverse primer (10 pmol/μL), 2 μL of the extracted DNA template, and 9.3 μL of nuclease-free water. The master mix contained MgCl₂ at a final concentration of 1.5 mM. The nucleotide sequences of the primers are shown in Table 1 in S1 File.

The PCR reaction was performed for 35 amplification cycles consisting of denaturation at 94 °C for 30 seconds, annealing at 62 °C for 30 seconds, and extension at 72 °C for 1 minute, using BioRad T100 Thermal Cycler [43]. The PCR products were electrophoresed on a 1.5% agarose gel in 1X Tris-acetate EDTA (TAE) buffer and stained with EtBr. Vivantis’ 100-bp DNA ladder was used as a molecular marker, and the Bio-Rad GelDoc EZ Gel imaging device visualised DNA bands under UV light.

### 2.5. 16S rRNA sequence analysis

Species identification of isolated *Aeromonas* was achieved using PCR amplification with the following primers: 27F: 5′-AGAGTTTGATCCTGGCTCAG-3′; 1392R: 5’-GGTTACCTTGTTACGACTT-3′ supplied by Macrogen Oligo. Each PCR reaction was prepared for 20 μL, including 10 μL of Add Taq 2X PCR mastermix (addbio, Korea), 0.25 μL of forward and reverse primers (10 pmol/μL), 2 μL of DNA template, and 7.5 μL of nuclease-free water. Amplification was performed on a Bio-Rad T100 thermal cycler for 35 cycles, denaturation was at 95 ºC, annealing at 55 ºC for 30 seconds, and extension at 72 ºC for 1 minute 30 seconds [44]. ExoSAP-IT^TM^ PCR Product Cleanup Reagent, made by Thermo Fisher Scientific, was used to remove excess primers and dNTPs from the PCR products. Then, purified products were cycle sequenced using the BigDyeTM Terminator v3.1 Cycle Sequencing Kit (Thermo Fisher Scientific) following the manufacturer’s instructions. The sequencing reaction consisted of 25 cycles of denaturation at 96 ºC for 10 seconds, annealing at 50 ºC for 5 seconds, and extension at 60 ºC for 4 minutes. These products were further purified using the D-Pure™ DyeTerminator Cleanup Kit. Sequencing runs were conducted in a 3500 Dx Genetic Analyzer, and raw sequencing data were analyzed using Sequencing Analysis Software v6.0 (Thermo Fisher Scientific). The quality of the obtained sequences was assessed using the Phred quality score, and high-quality sequences were aligned and compared to reference sequences in the NCBI GenBank database using the BLASTn tool for species identification.

After analysis, all the sequences were submitted to the NCBI GenBank. The accession numbers for the full nucleotide sequence of 16S rRNA from the fish *Aeromonas* spp. strains that were submitted to GenBank are displayed in Table 2 and Table 2 in S1 File.

**Table 2:** Taxonomic relationships of the samples were determined by sequencing their 16S rRNA genes.

Isolate ID	Accession numbers	Taxonomy annotation hierarchy	Sequence analysis with BLAST			
			Sequence analysis with BLAST	Query cover (%)	Percent identity (%)	E value
**2C2**	OR603963	**Genus:** *Aeromonas*	*A. veronii*	100	99.64	0
**2C7**	OR603964		*A. veronii*	100	97.64	0
**2C8**	OR603965	**Family:** Aeromonadaceae	*A. jandaei*	100	99.93	0
**3AERO**	OR603966		*A. hydrophila*	100	99.93	0
**3C2**	OR603967	**Order:** Aeromonadales	*A. veronii*	100	98.93	0
**3C4**	OR603968		*A. veronii*	100	99.86	0
**3C6**	OR603969	**Class:** Gammaproteobacterias	*A. veronii*	100	98.49	0
**3C7**	OR603970		*A. veronii*	100	99.07	0
**4C2**	OR603971	**Phylum:** Pseudomonadota	*A. veronii*	100	99.93	0
**5C2**	OR603972		*A. veronii*	100	99.73	0
**5C6**	OR603973		*A. veronii*	100	99.86	0
**5C9**	OR603974		*A. bivalvium*	100	99.93	0
**5C10**	OR603975		*A. hydrophila*	100	99.5	0
**6C6**	OR116112		*A. veronii*	100	100	0
**6M2**	OR603976		*A. veronii*	100	99.61	0
**10X2**	OR603977		*A. jandaei*	100	100	0
**11M1**	OR603978		*A. veronii*	100	99.75	0
**13X1**	OR603979		*A. veronii*	100	99.93	0
**14M2**	OR603980		*A. veronii*	100	99.93	0
**14M4**	OR603981		*A. veronii*	100	100	0
**15T1**	OR603982		*A. veronii*	100	99.02	0
**16C7**	OR603983		*A. caviae*	100	99.78	0

The obtained 16S rRNA gene sequences were compared with reference sequences available in the NCBI GenBank database using the BLASTn algorithm to determine species identity. Taxonomic identification was assigned based on the highest sequence similarity, query coverage, and E-value obtained from BLAST results. The murkiness surrounding the taxonomic categorization of enrichment culture clones, uncultured bacteria, and unclassified bacteria led to their exclusion.

### 2.6. Phylogenetic data analysis

The quality of the 16S rRNA gene sequences from *Aeromonas* isolates was assessed, and a consensus sequence was computed from the forward and reverse sequences using MEGA version 11. Muscle, a method found in version 11 of the software Molecular Evolutionary Genetics Analysis (MEGA), was used to align the 16S rRNA gene sequences of the *Aeromonas* isolates with the strains that scored highest in the BLAST results. A neighbor-joining phylogenetic analysis of the aligned sequences was conducted with the neighbour-joining method and 1000 iterations for bootstrap support using MEGA version 11 software [45–47].

### 2.7. Antibiotic susceptibility testing

All the isolates of *Aeromonas* (n = 22) were tested for antimicrobial drug resistance. Isolates were cultivated overnight in TSB at 37 °C for 24 h. Then a standardized bacterial inoculum was used for antimicrobial susceptibility testing, representing a bacterial suspension of approximately 1.5 x 10^8 CFU/mL, adjusting the turbidity of the broth to a 0.5 McFarland standard [48,49].

**Fig 3:**
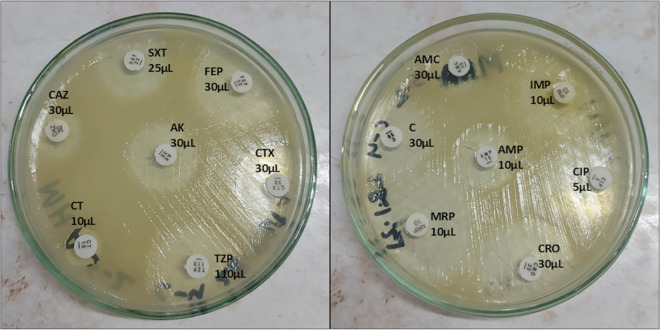
Antimicrobial susceptibility test result plates of *Aeromonas* isolates.

The inoculum was spread onto Mueller-Hinton agar (MHA) using a sterile swab stick. Then we used standard antimicrobial disks (Oxoid, UK) contained a range of antimicrobials at specific concentrations, which included Ampicillin (10 μg), Amoxicillin-clavulanic acid (30 μg), Piperacillin-Tazobactam (110 μg), Amikacin (30 μg), Ciprofloxacin (5 μg), Cefotaxime (30 μg), Cefepime (30 μg), Ceftazidime (30 μg), Ceftriaxone (30 μg), Chloramphenicol (30 μg), Imipenem (10 μg), Meropenem (10 μg), and Sulfamethoxazole/Trimethoprim (25 μg) on MHA plates. Then the plates were incubated for 24 h at 37 °C (Fig 3). Following incubation, the zones of inhibition around each antimicrobial disk were interpreted according to the Clinical and Laboratory Standards Institute (CLSI M100, 30th edition, 2020) guidelines using breakpoints established for *Enterobacterales*, as CLSI does not provide specific interpretive criteria for *Aeromonas* spp. This study used first-generation, second-generation, third-generation, and fourth-generation antimicrobials with narrow and broad-spectrum activity to observe the variety of organisms’ susceptibility to the antimicrobials of different generations, which are mostly used.

### 2.8. Detection of virulence genes

Amplification of the genes encoding cytotoxic enterotoxin (*act*), hemolysin (*hlyA*), heat-labile cytotoxic enterotoxin (*alt*), flagellin (*laf*), Shiga toxins (*stx-1* and *stx-2*), type III secretion system (*ascF-ascG*), allatostatin (*ast*), and aerolysin (*aerA*) was performed using polymerase chain reaction (PCR) from each bacterial isolate. The primer sets specific to these genes are detailed in Table 1 in S1 File. Amplification of the nine virulence genes was carried out using identical thermocycling techniques as the *gyrB* gene, except for the use of different annealing temperatures. The gel electrophoresis process was similar to section 2.4.

### 2.9. Statistical analysis

All the statistical analyses were done using R version 4.4.1. The multiple antibiotic resistance (MAR) index was computed per isolate as the number of resistant outcomes divided by the number of antibiotics tested. Multidrug resistance (MDR) was defined as non-susceptibility to ≥3 antimicrobial classes. Differences in resistance burden among fish groups were tested using a binomial GLM (logit) with p < 0.05. Antibiotic susceptibility outcomes were recorded as susceptible (S), intermediate (I), or resistant (R). Thus, for regression analysis, resistance burden was summarized for each isolate as the number of resistant events (“R”) out of 13 antibiotics and analyzed using GLM with a binomial error distribution and a logit link. Confidence intervals (95% CI) for proportions were in R using the exact binomial (Clopper–Pearson) method in R programming (**R codes are available in** S1 File).

## 3. Results

### 3.1. Isolation and identification of the *Aeromonas* isolates

**Fig 4:**
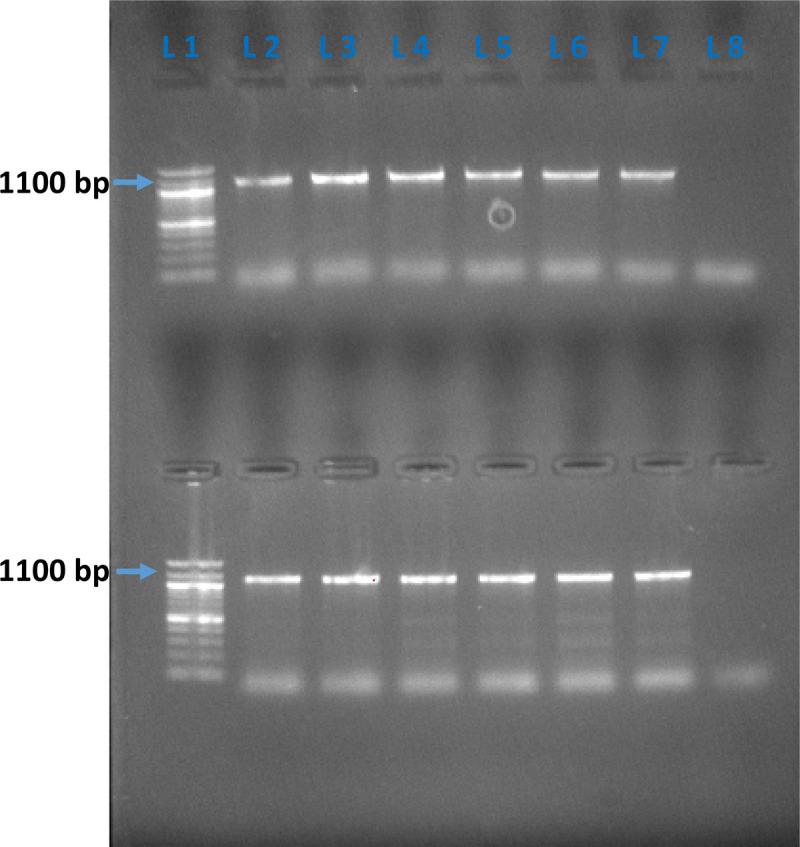
Agarose gel electrophoresis of PCR products targeting the *gyrB* gene for *Aeromonas* confirmation: DNA ladder in line 1 (L1) and negative control line 8 (L8).

Sixty-four out of 94 isolates (64/94, 68%) were presumptively identified as belonging to the genus *Aeromonas* based on phenotypic and biochemical tests. Of these, 22 (22/64, 34.4%) isolates were confirmed as *Aeromonas* spp*.* by *gyrB* gene detection, producing a distinct ~1100 bp amplicon on gel electrophoresis (Fig 4).

### 3.2. Taxonomic analysis

16S rRNA gene sequences were analyzed for phylogeny and homology, which determined the molecular taxonomy of *Aeromonas* strains. Species-level identification was assigned based on ≥98% sequence identity, 100% query coverage, and an E-value of 0 (zero) obtained from BLASTn comparison with reference sequences in the NCBI GenBank database (Table 2). A 16S rRNA gene sequence identity above 98% is commonly used as an initial criterion for species-level identification within the genus *Aeromonas*, supporting the preliminary taxonomic assignments made in this study [50].

### 3.3. Prevalence of isolates and phylogenetic analysis

**Fig 5:**
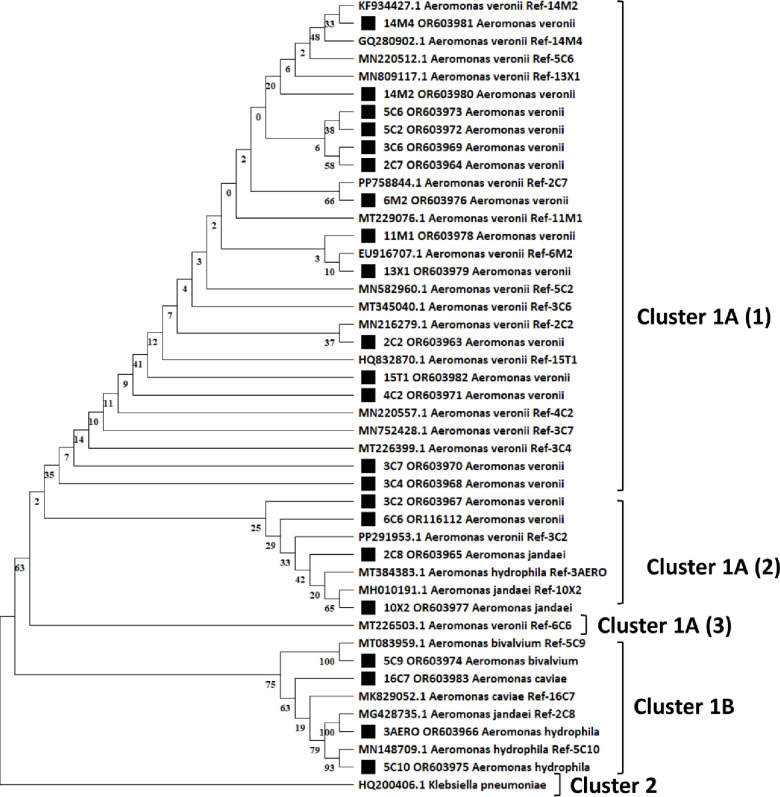
Neighbor-joining phylogenetic tree based on partial 16S rRNA gene sequences showing relationships between *Aeromonas* isolates obtained in this study and reference strains retrieved from GenBank. Bootstrap values were calculated from 1000 replicates and are shown at branch nodes.

**Fig 6:**
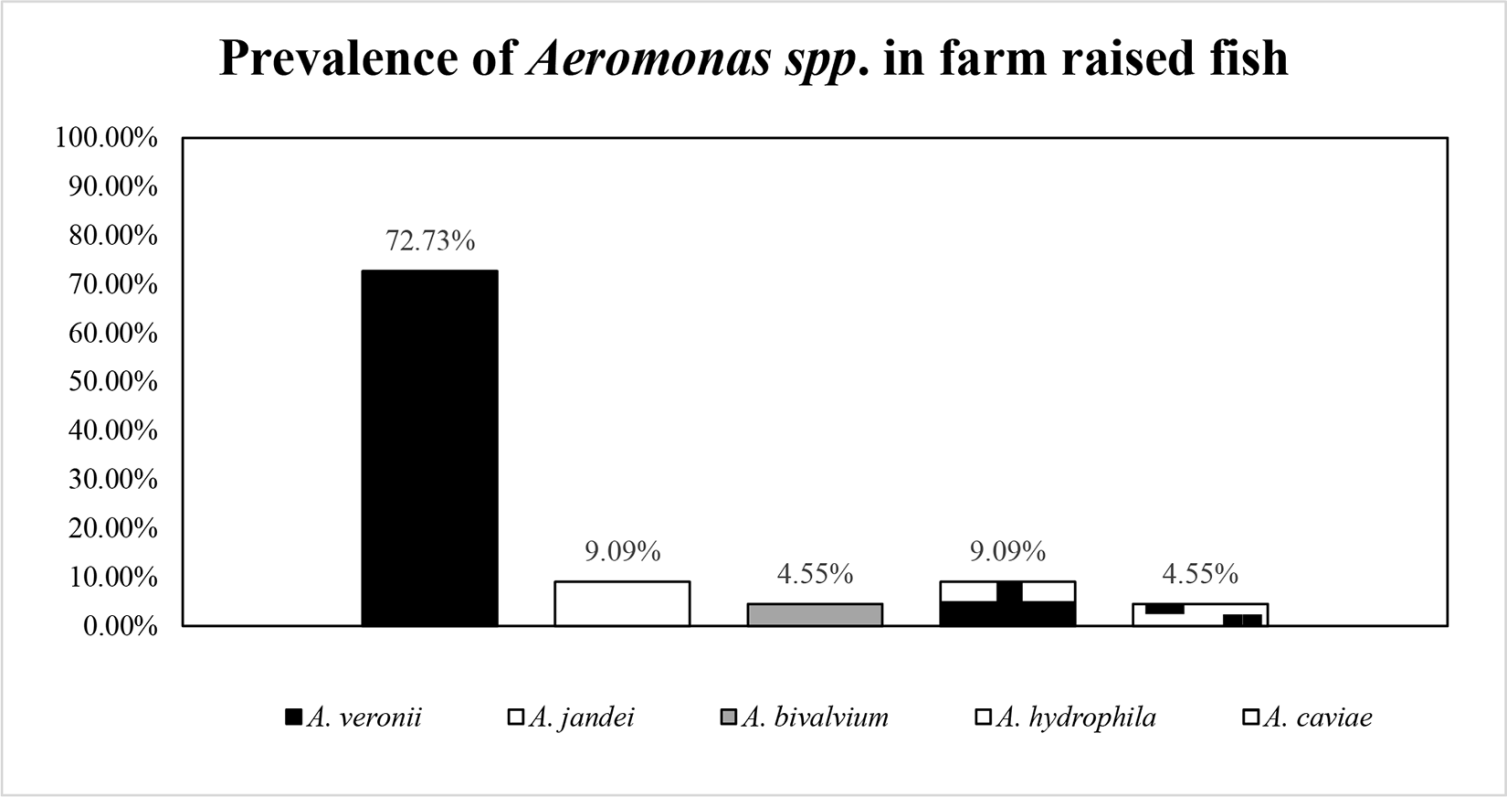
The 16S rRNA gene sequence was used to characterize the prevalence of *Aeromonas* spp. present in farm-raised fish.

A total of 22 *Aeromonas* strains were identified using 16S rRNA gene sequencing, of which 32% were isolated from *O. niloticus* and 68% from *L. rohita.* These 22 presumed *Aeromonas* isolates were divided into five species based on an unrooted Tamura-Nei neighbor-joining (NJ) tree, which included 22 reference strains with partial sequences (Fig 5). *A. veronii* was the predominant species (72.73%, 95% CI: 49.8–89.3), followed by *A. hydrophila* (9.09%, 95% CI: 1.1–29.2), *A. jandaei* (9.09%, 95% CI: 1.1–29.2), *A. caviae* (4.55%, 95% CI: 0.1–22.8), and *A. bivalvium* (4.55%, 95% CI: 0.1–22.8), as shown in Fig 6.

Phylogenetic analysis of the 16S rRNA gene sequences showed that the isolates clustered with their corresponding reference strains, confirming species-level identification (Fig 5). The phylogenetic tree formed two main clusters: Cluster 1 contained all *Aeromonas* isolates and reference sequences, whereas Cluster 2 consisted of the outgroup *Klebsiella pneumoniae*. Within Cluster 1, *A. veronii* isolates formed subcluster 1A, while *A. bivalvium*, *A. caviae*, *A. jandaei*, and *A. hydrophila* grouped within subcluster 1B.

### 3.4. Antimicrobial susceptibility profiling and MAR indexing

All the *Aeromonas* isolates were highly resistant to ampicillin (95%) and amoxicillin-clavulanic acid (86%). Additionally, approximately 27% of the isolates demonstrated resistance to the β-lactam antibiotic ceftazidime (Table 3). On the other hand, the remaining isolates were found to be sensitive to ciprofloxacin, cefepime, ceftriaxone, chloramphenicol, and sulfamethoxazole/trimethoprim. The multiple antibiotic resistance (MAR) index (Table 4) was calculated for each isolate, and isolates with a MAR index >0.2 are considered to originate from environments with high antibiotic exposure [51]. The MAR index values ranged from 0.07 to 0.69; 16 isolates (16/22, 68.2%) were found to be positive for MAR status. Among *L. rohita* -derived isolates, resistance was observed to 1–5 antibiotics, with MAR index ranging from 0.07 to 0.38. In contrast, isolates derived from the *O. niloticus* showed higher resistance burden, showing resistance to 3–9 antibiotics and MAR index values ranging from 0.23 to 0.69. Overall, MAR-positive isolates were more frequent among *O. niloticus* (85.7%) than *L. rohita* (60.0%), indicating a greater exposure of Tilapia-associated isolates to antimicrobial selective pressure.

**Table 3:** This table represents the results of the antibiotic susceptibility test of *Aeromonas* spp. performed by the disc diffusion method interpreted as susceptible (S), intermediate (I), and resistant (R).

Antimicrobial agents	Concentration (µg)	Bacterial isolates (22 isolates)					
		R		I		S	
		No. of isolates	%	No. of isolates	%	No. of isolates	%
Ampicillin (AMP)	10	21	95	1	5	0	0
Amoxicillin-clavulanic acid (AMC/AML)	30	18	82	2	9	2	9
Piperacillin-Tazobactum (TZP)	10	3	13	1	4	18	81
Amikacin (AK)	30	2	9	4	18	16	73
Ciprofloxacin (CIP)	5	2	9	0	0	20	91
Cefotaxime (CTX)	30	1	5	0	0	21	95
Cefepime (FEP)	30	1	5	4	18	17	77
Ceftazidime (CAZ)	30	6	27	5	23	11	50
Ceftriaxone (CRO)	30	3	14	0	0	19	86
Chloramphenicol (C)	30	0	0	0	0	22	100
Imipenem (IMP)	10	8	36	8	36	6	27
Meropenem (MEM)	10	10	45	2	9	10	45
Sulfamethoxazole/ trimethoprim (SXT)	25	4	18	1	4	17	77

**Table 4:** MAR index of the isolates from *Oreochromis niloticus* and *Labeo rohita.*

Isolate ID	Fish	Antimicrobial resistance pattern	No. of antibiotics to which the isolate was resistant	MAR index	MAR status
2C2	*Rui*	Ampicillin, Amoxicillin-clavulanic acid, Piperacillin-Tazobactum, Imipenem, Meropenem,	5	0.38	Yes
2C7	*Rui*	Ampicillin, Amoxicillin-clavulanic acid, Meropenem	3	0.23	Yes
2C8	*Rui*	Ampicillin, Amoxicillin-clavulanic acid	2	0.15	No
3AERO	*Rui*	Ampicillin, Ciprofloxacin, Ceftazidime	3	0.23	Yes
3C2	*Rui*	Ampicillin, Amoxicillin-clavulanic acid, Ceftriaxone, Meropenem	4	0.31	Yes
3C4	*Rui*	Ampicillin, Amoxicillin-clavulanic acid, Ceftazidime	3	0.23	Yes
3C6	*Rui*	Ampicillin, Amoxicillin-clavulanic acid, Ceftazidime, Meropenem	4	0.31	Yes
3C7	*Rui*	Ampicillin, Amoxicillin-clavulanic acid, Ceftazidime, Meropenem	4	0.31	Yes
4C2	*Tilapia*	Ampicillin, Amoxicillin-clavulanic acid, Meropenem	3	0.23	Yes
5C2	*Rui*	Ampicillin, Amoxicillin-clavulanic acid	2	0.15	No
5C6	*Rui*	Ampicillin, Amoxicillin-clavulanic acid, Imipenem, Meropenem	4	0.31	Yes
5C9	*Rui*	Amoxicillin-clavulanic acid	1	0.07	No
5C10	*Rui*	Ampicillin, Amoxicillin-clavulanic acid, Meropenem	3	0.23	Yes
6C6	*Rui*	Ampicillin, Sulfamethoxazole/ trimethoprim	2	0.15	No
6M2	*Rui*	Ampicillin, Sulfamethoxazole/ trimethoprim	2	0.15	No
10X2	*Tilapia*	Ampicillin, Amoxicillin-clavulanic acid, Imipenem	3	0.23	Yes
11M1	*Rui*	Ampicillin, Amoxicillin-clavulanic acid	2	0.15	No
13X1	*Tilapia*	Ampicillin, Ciprofloxacin, Ceftazidime, Imipenem	4	0.31	Yes
14M2	*Tilapia*	Ampicillin, Amoxicillin-clavulanic acid, Piperacillin-Tazobactum, Amikacin, Imipenem, Meropenem	6	0.46	Yes
14M4	*Tilapia*	Ampicillin, Amoxicillin-clavulanic acid, Piperacillin-Tazobactum, Amikacin, Imipenem	5	0.38	Yes
15T1	*Tilapia*	Ampicillin, Amoxicillin-clavulanic acid, Cefotaxime, Cefepime, Ceftazidime, Ceftriaxone, Imipenem, Meropenem, Sulfamethoxazole/ trimethoprim	9	0.69	Yes
16C7	*Rui*	Ampicillin, Amoxicillin-clavulanic acid, Ceftriaxone, Imipenem, Sulfamethoxazole/ trimethoprim	5	0.38	Yes

MAR index >0.2 = MAR status “Yes”, MAR index <0.2 = MAR status “No” [51].

### 3.5. Fish species–specific differences in antibiotic resistance

A binomial generalized linear model (GLM) was used to evaluate the burden of antibiotic resistance in *Aeromonas* isolated from two fish species. We used *L. rohita* isolates as the reference group; the binomial GLM intercept (β = −1.177) represents the baseline resistance burden across the 13 antibiotics tested, corresponding to a resistance probability of ~3.1 resistant antibiotics per *L. rohita* isolate. In comparison, *O. niloticus* isolates had significantly higher odds of resistance burden than those from *L. rohita* (β = 0.707 ± 0.284 SE; z = 2.487; *p* < 0.05) (Table 5), indicating approximately a two-fold increase in the likelihood of resistance among *O. niloticus* isolates. The probability of ~5.0 resistant antibiotics out of 13 antibiotics per isolate.

**Table 5:** Binomial generalized linear model evaluating the effect of fish species on antibiotic resistance.

Predictor	Estimate (log-odds)	Std. Error	z value	p-value	Odds Ratio
Intercept *(L. rohita)*	−1.1771	0.1634	−7.204	<0.0001	0.31 (0.22–0.42)
*O. niloticus (vs L. rohita)*	0.7071	0.2844	2.487	<0.05	2.03 (1.16–3.54)

Statistical significance was set at p ≤ 0.05.

Model fit: Null deviance = 23.77 (df = 21), Residual deviance = 17.71 (df = 20), AIC = 81.75.

### 3.6. Assessment of virulence genes

**Fig 7:**
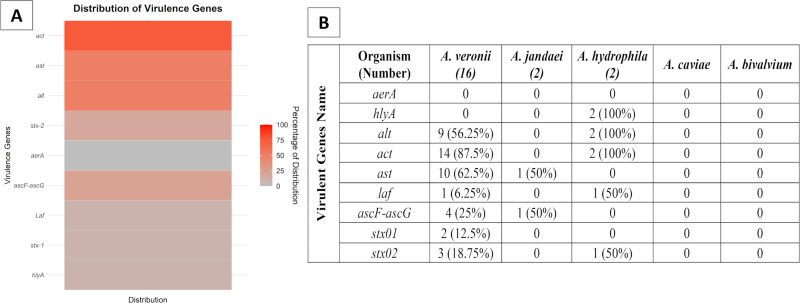
A. Heat-map showing the distribution of virulence gene-harboring *Aeromonas* strains, B. Overall number of virulence genes in each isolate.

All the isolates were screened for the presence of nine specific genes (*aerA, act, ast, hlyA, alt, laf, stx-1, stx-2*, and *ascF-ascG*) through PCR. The distribution of virulence genes within fish-derived *Aeromonas* isolates is as follows: The cytotoxic enterotoxin gene *act* was the most frequently detected virulence gene, found in 72.72% (16/22) of the isolates. The *ast* gene, linked to a heat-labile cytotonic enterotoxin, and the *alt* gene, associated with a secreted heat-stable enterotoxin, were present in 50% (11/22) of the isolates. The *stx-2* gene was identified in 18.18% of the isolates, and the *ascF-ascG* gene in 22.73% (5/22) of the isolates. Meanwhile, the *laf* gene, the *stx-1* gene, and the *hlyA* gene were detected in 9.09% of the isolates. Notably, the *aerA* gene was not found in any of the *Aeromonas* isolates after PCR (Fig 7).

## 4. Discussion

The expansion of aquaculture and the intensification of farming practices can increase the risk of bacterial diseases in farmed fish populations [52,53]. Notably, bacterial hemorrhagic septicemia and epizootic ulcerative syndrome (EUS) caused by *Aeromonas* spp. has become a significant cause of disease outbreaks and mortality in tilapia farming worldwide [54–57].

Assessment of *Aeromonas* spp. prevalence is challenged by ongoing taxonomic revisions and variability in diagnostic methodologies. Reliance on 16S rRNA gene sequencing alone frequently fails to resolve closely related species, such as *A. veronii* and *A. sobria* [58]. Although the *gyrB* gene was used for molecular confirmation of *Aeromonas* isolates in this study, the phylogenetic tree was constructed using 16S rRNA gene sequences alone. A concatenated phylogenetic analysis using multiple housekeeping genes would provide stronger taxonomic resolution for closely related *Aeromonas* species. Thus, advanced molecular approaches, including multilocus sequence typing (MLST) and sequencing of housekeeping genes, are recommended for more accurate species identification.

In the present study, five *Aeromonas* species were identified, namely *A. veronii*, *A. jandaei, A. hydrophila, A. caviae,* and *A. bivalvium*. Out of these, *A. veronii* was the most prevalent (72.72%) in consistent with previous findings from Bangladesh [59]. This finding is noteworthy because severe *Aeromonas* infections, especially bacteremia caused by *A. veronii and A. hydrophila,* have been linked to high case fatality rates (33–56%) in humans [60]. Potential routes of human exposure include the consumption of contaminated fish or water and contact of open wounds with contaminated aquatic environments. The predominance of *A. veronii* in the present study also supported by reports from Tanzania, China and Malaysia showing similarly high prevalence of *A. veronii* in healthy fish [61–63]. Moreover, the widespread distribution of *A. veronii* across diverse hosts, including humans, aquatic animals, food, and the environment, underscores its significance as an emerging pathogen in Bangladeshi aquaculture [16]. By contrast, the comparatively low prevalence of *A. hydrophila* differs from reports in Vietnam, Malaysia, and Egypt, which may reflect geographic variation in *Aeromonas* species distribution [64,65].

Pathogenicity in *Aeromonas* spp. is contributed by several virulence factors, including enterotoxins (*act, alt, ast*) and hemolysins (*hlyA, aerA*) [66]. Interestingly, the *aerA* gene, which is commonly associated with highly virulent *A. hydrophila* strains and severe disease, was not detected in any of the isolates [67–70]. Previous studies have reported high prevalence of the *hlyA* gene among *Aeromonas* isolates [71–73]; however, in the present study, *hlyA* was detected exclusively in *A. hydrophila* isolates and was absent in all other species. The absence of these virulence-associated genes may be attributed to the fact that isolates were obtained from clinically healthy fish.

The high prevalence of the *act* gene among *A. veronii* isolates in this study is consistent with previous reports highlighting its association with *Aeromonas* pathogenicity, whereas the lower detection of *ast*, *alt*, and other virulence genes may reflect strain-specific variation reported in earlier studies [31,74–77]. However, because all fish sampled in this study were clinically healthy, the detection of virulence-associated genes should not be interpreted as evidence of active disease. These genes only indicate the potential pathogenic capability of the isolates, and further phenotypic or infection studies would be required to confirm their role in disease development. Flagellar genes are essential for motility and colonization; however, only 9.1% of isolates carried the *laf* gene, much lower than rates reported elsewhere [78–80]. This variability highlights the complex interactions among environmental, genetic, and methodological factors influencing *Aeromonas* spp. pathogenicity.

Antimicrobial resistance (AMR) among *Aeromonas* isolates presents a significant concern. The majority of the isolates exhibited resistance to ampicillin (95%) and amoxicillin-clavulanic acid (82%), consistent with the prevalence of widespread occurrence of chromosomally encoded β-lactamase genes [81]. No resistance to chloramphenicol was observed, indicating its continued potential as a therapeutic agent, though ongoing monitoring is warranted. Resistance to ciprofloxacin was low (9%), but resistance rates to carbapenems, imipenem (36%), and meropenem (45%) were much higher than previously reported (0.5–7.7%) [82]. This study also demonstrated a high percentage of *Aeromonas* isolates (68%) with a MAR index of more than 0.2, which indicates that the *Aeromonas* strains originated from a high-risk source of contamination [83]. Overall, the antimicrobial resistance patterns and multidrug resistance observed in this study highlight the presence of resistant *Aeromonas* isolates in aquaculture-associated fish. This study focused on phenotypic antimicrobial resistance patterns; however, specific antimicrobial resistance genes such as *blaTEM*, *blaSHV*, *blaCTX-M*, *cphA*, *qnr* genes, *tet* genes, or *sul* genes were not investigated. The coexistence of virulence-associated genes and antimicrobial resistance in the same isolates may increase overall public health concern; however, no direct relationship between these traits was investigated in this study.

In summary, our findings underscore the necessity for enhanced surveillance and monitoring of *Aeromonas* spp. in aquaculture, judicious antimicrobial use, and the development of preventive strategies such as vaccination and improved management practices. Addressing these challenges is crucial for sustainable aquaculture, food safety, and public health in Bangladesh and other developing countries.

## 5. Limitations

This study provides important epidemiological insights into *Aeromonas* spp. in Bangladesh; however, several limitations should be acknowledged. Sampling was limited to a single region and season, which may not reflect broader geographic or temporal variation. Isolation was performed using MacConkey agar, a non-selective medium that may affect recovery efficiency. Antimicrobial susceptibility was interpreted using *Enterobacterales* breakpoints due to the lack of CLSI standards for *Aeromonas*, which may introduce interpretive limitations. Phylogenetic analysis relied solely on 16S rRNA gene sequences, which have limited discriminatory power for closely related species. PCR detection of virulence genes indicates only their presence and does not confirm gene expression or toxin production; moreover, all fish sampled were clinically healthy, so these findings do not indicate disease. Additionally, antimicrobial resistance genes were not investigated, limiting insights into resistance mechanisms. Future studies should include broader sampling, incorporate multilocus or whole-genome approaches, and include both healthy and diseased fish to better understand pathogenicity and resistance.

## 6. Conclusion

This study molecularly identified *Aeromonas* spp. from farm-raised *O. niloticus* and *L. rohita* in Noakhali, Bangladesh, revealing *A. veronii* as the predominant species. High prevalence of key virulence genes and multidrug resistance, especially against β-lactams, including carbapenems, indicates significant threats to aquaculture biosecurity and public health. These findings highlight the need for strengthened surveillance, responsible antimicrobial use, and improved farm management practices.

## Supporting information

S1 File Supplementary S1. (DOCX)

S2 File Supplementary S2- Sensitivity data of all isolates. (XLSX)

## Abbreviations

CLSI: Clinical and Laboratory Standards Institute
EUS: Epizootic Ulcerative Syndrome
MAS: Motile *Aeromonas* Septicemia
MDR: Multidrug-Resistant
MHA: Mueller Hinton Agar
MIU: Motility Indole Urea
PBS: Phosphate Buffered Saline
PCR: Polymerase Chain Reaction
TAE: Tris-acetate-EDTA
TSB: Tryptic Soy Broth
TSI: Triple Sugar Iron
