# Supplementary material for: Prevalence, potential virulence genes, and antimicrobial resistance of Aeromonas spp. in farm-raised Oreochromis niloticus and Labeo rohita in Noakhali, Bangladesh
Source: PLoS One. 2026 Apr 28;21(4):e0347577. doi: 10.1371/journal.pone.0347577 (PMC13123944; doi:10.1371/journal.pone.0347577)
Supplement: S1 File — (DOCX) [file pone.0347577.s001.docx]

**Supplementary Table 1.** Primer sequences are used for the polymerase chain reaction (PCR) to amplify gyrB gene, and virulence genes of *Aeromonas*.

| **Target Genes** | **Primer Sequences (5'**-**3')** | **Product Size (bp)** | **Optimal Annealing Temperature (ºC)** |  |
| --- | --- | --- | --- | --- |
|  |  |  |  |  |
|  |  |  |  |  |
| GyrB | **F** TCCGGCGGTCTGCACGGCGT | 1100 | 62 |  |
|  | **R** TTGTCCGGGTTGTACTCGTC |  |  |  |
| aerA | **F** GCTGAACCCATCTATCCTG | 301 | 57 |  |
|  | **R** TTTCTCCGGTAACAGGATTG |  |  |  |
| hlyA | **F** GGCCGGTGGCCCGAAGATACGGG | 600 | 72 |  |
|  | **R** GGCGGCGCCGGACGAGACGGG |  |  |  |
| Alt | **F** CCATCCCCAGCCTTTACGCCAT | 338 | 66 |  |
|  | **R** TTTCACCGAGGTGACGCCGT |  |  |  |
| ast | **F** ATGCACGCACGTACCGCCAT | 260 | 62 |  |
|  | **R** ATCCGGTCGTCGCTCTTGGT |  |  |  |
| Laf | **F** GGTCTGCGCATCCAACTC | 550 | 57 |  |
|  | **R** GCTCCAGACGGTTGATG |  |  |  |
| ascF – ascG | **F** ATGAGGTCATCTGCTCGCGC | 789 | 60 |  |
|  | **R** GGAGCACAACCATGGCTGAT |  |  |  |
| Stx-1 | **F** ATAAATTGCCATTCGTTGACTAC | 180 | 57 |  |
|  | **R** AGAACGCCCACTGAGATCATC |  |  |  |
| Stx-2 | **F** GGCACTGCTTGAAACTGCTCC | 255 | 56 |  |
|  | **R** TCGCCAGTTATCTGACATTCTG |  |  |  |
| Act | **F** ATCGTCAGCGACAGCTTCTT | 500 | 60 |  |
|  | **R** CTCATCCCTTGGCTTGTTGT |  |  |  |

**Supplementary Table 2.** Accession numbers of Aeromonas isolated from farm-raised *O.* niloticus and L. rohita.

| **Isolate ID** | **Accession Number** | **Isolate ID** | **Accession Number** |
| --- | --- | --- | --- |
| 2C2 | OR603963 | 5C9 | OR603974 |
| 2C7 | OR603964 | 5C10 | OR603975 |
| 2C8 | OR603965 | 6C6 | OR116112 |
| 3AERO | OR603966 | 6M2 | OR603976 |
| 3C2 | OR603967 | 10X2 | OR603977 |
| 3C4 | OR603968 | 11M1 | OR603978 |
| 3C6 | OR603969 | 13X1 | OR603979 |
| 3C7 | OR603970 | 14M2 | OR603980 |
| 4C2 | OR603971 | 14M4 | OR603981 |
| 5C2 | OR603972 | 15T1 | OR603982 |
| 5C6 | OR603973 | 16C7 | OR603983 |

**Supplementary Figure 1.** Representative picture of the whole fish of *Labeo rohita* and *Oreochromis niloticus* used in this study.


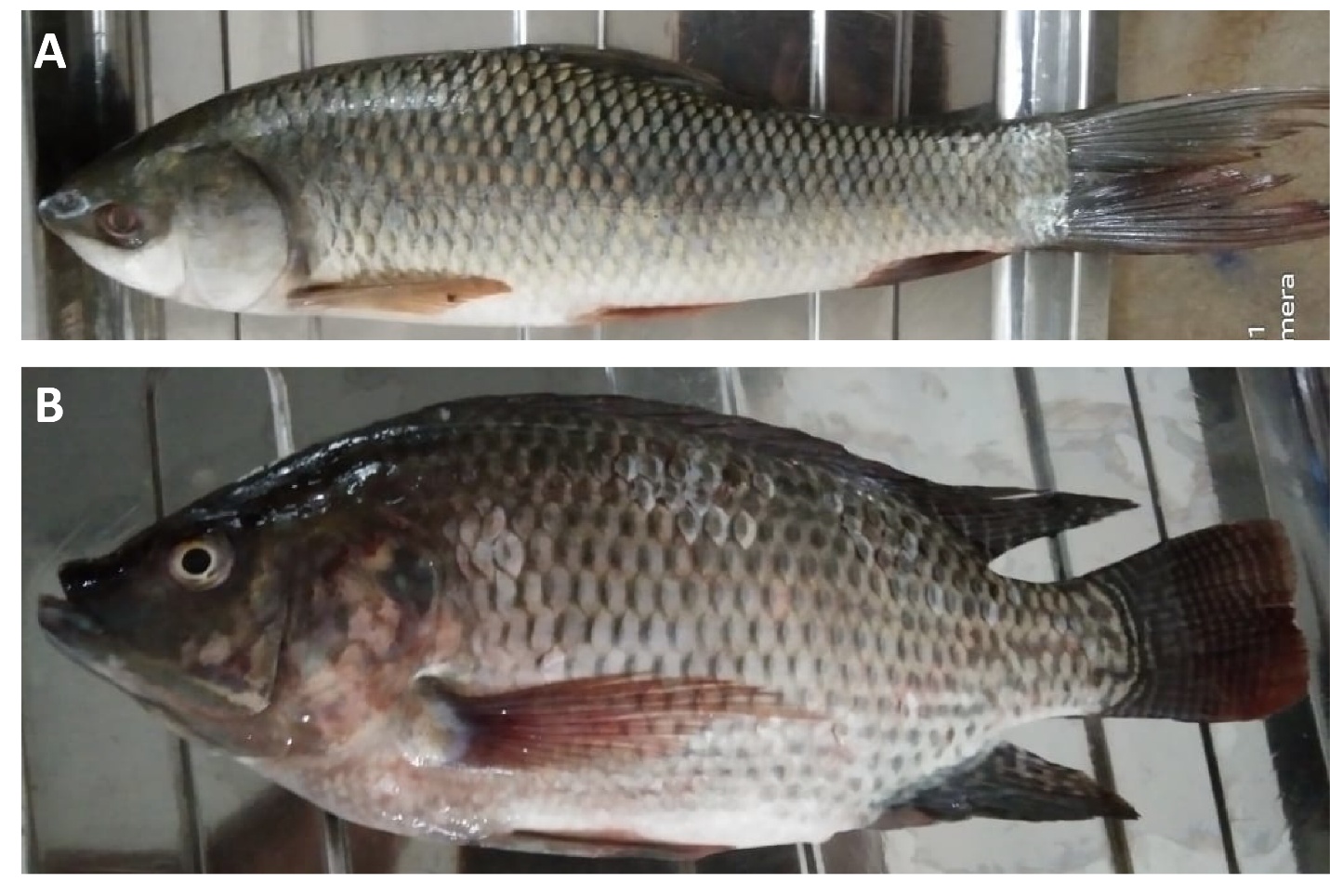


**R programming code for regression analysis:**

library(dplyr)

library(tidyr)

library(ggplot2)

library(readr)

library(mgcv)

library(writexl)

**data = Supplementary_S2**

**#First clean the data and make a new excel sheet for analysis**

data$Colistin <- NULL

**#now make a new data sheet**

clean_data <- data %>%

pivot_longer(cols = -c(Isolate, Fish),

names_to = "antibiotic",

values_to = "result") %>%

mutate(

result = toupper(trimws(result)),

nonsus = result %in% c("R","I"),

resist = result == "R")

**clean_data <- clean_data %>% filter(antibiotic != "Aeromonas spp")**

**#Antibiotic resistance table**

amr_summary <- clean_data %>%

mutate(

result = toupper(trimws(result)),

result = na_if(result, ""),

valid = result %in% c("R","I","S","SDD"),

nonsus = result %in% c("R","I","SDD")

) %>%

filter(valid) %>%

group_by(antibiotic) %>%

summarise(

n_tested = n(),

R = sum(result == "R"),

I = sum(result == "I"),

S = sum(result == "S"),

SDD = sum(result == "SDD"),

Non_susceptible = sum(nonsus),

pct_R = 100 * R / n_tested,

pct_I = 100 * I / n_tested,

pct_S = 100 * S / n_tested,

pct_NS = 100 * Non_susceptible / n_tested,

.groups = "drop"

) %>%

arrange(desc(pct_NS))

**amr_summary**

**write_xlsx(amr_summary, "amr_summary.xlsx")**

**#Multiple antibiotic resistance (MAR) index**

mar <- clean_data %>%

mutate(result = toupper(trimws(result))) %>%

group_by(`Isolate`, Fish) %>%

summarise(

total = n(),

n_R = sum(result == "R", na.rm = TRUE),

MAR_R = n_R / total,

.groups = "drop"

)

**mar**

**as.data.frame(mar)**

**write_xlsx(mar, "mar.xlsx")**

**#Multidrug resistance profile of fish isolates**

class_map <- tibble::tribble(

~antibiotic, ~class,

"Ampicillin", "Penicillins",

"Amoxicillin-clavulanic acid", "Penicillins",

"Piperacillin-Tazobactum", "Penicillins",

"Cefotaxime", "Cephalosporins",

"Cefepime", "Cephalosporins",

"Ceftazdime", "Cephalosporins",

"Ceftriaxone", "Cephalosporins",

"Imipenem", "Carbapenems",

"Meropenem", "Carbapenems",

"Amikacin", "Aminoglycosides",

"Ciprofloxacin", "Fluoroquinolones",

"Chloramphenicol", "Phenicols",

"Sulfamethoxazole/ trimethoprim", "Folate inhibitors",

"Colistin", "Polymyxins"

)

**#multidrug resistance**

mdr <- clean_data %>%

left_join(class_map, by = "antibiotic") %>%

group_by(`Isolate`, Fish, class) %>%

summarise(class_res = any(nonsus), .groups = "drop") %>%

group_by(`Isolate`, Fish) %>%

summarise(

classes_resistant = sum(class_res),

MDR = classes_resistant >= 3,

.groups = "drop"

)

**mdr**

**as.data.frame(mdr)**

**write_xlsx(mdr, "mdr.xlsx")**

**#Significance of antibiotic resistance vs Fish**

**#now create the data table with Fish, isolates, no of antibiotics each isolates**

**#resistant to and the proportion out of 13 antibiotics**

res_burden <- clean_data %>%

mutate(result = toupper(trimws(result))) %>%

group_by(`Isolate`, Fish) %>%

summarise(

antibiotic = n(),

n_R = sum(result == "R", na.rm = TRUE),

prop_R = n_R *100 / antibiotic,

.groups = "drop"

) %>%

arrange(desc(n_R))

**#now fit the binomidal generalized linear model**

glm_bin <- glm(

cbind(n_R, antibiotic - n_R) ~ Fish,

family = binomial,

data = res_burden

)

**summary(glm_bin)**

**#now compute odds ratio and confidence intervals and probability for the intercept and reference**

**coefficients <- summary(glm_bin)$coef**

Odds_ratio_data <- data.frame(

term = rownames(coefficients),

estimate = co[, "Estimate"],

se = co[, "Std. Error"],

z = co[, "z value"],

p = co[, "Pr(>|z|)"]

)

Odds_ratio_data$OR <- exp(Odds_ratio_data$estimate)

Odds_ratio_data$CI_low <- exp(Odds_ratio_data$estimate - 1.96 * Odds_ratio_data$se)

Odds_ratio_data$CI_high <- exp(Odds_ratio_data$estimate + 1.96 * Odds_ratio_data$se)

**Odds_ratio_data**

**#now calculate the probability**

**#Probability for the reference group (e.g., L. rohita)**

p_ref <- plogis(coef(glm_bin)[1])

p_ref

#probability of resistant per isolate

p_ref = p_ref*13 #no of antibiotic tested

p_ref

**#Probability for Tilapia**

eta_tilapia <- coef(glm_bin)[1] + coef(glm_bin)["FishTilapia"]

p_tilapia <- plogis(eta_tilapia)

p_tilapia

**#probability of resistant per isolate**

p_tilapia = p_tilapia*13

p_tilapia

**R programming code for exact binomial (Clopper–Pearson) method**

# Total number of isolates

total_isolates <- 22

# Species counts

species_counts <- c(

"A_veronii" = 16,

"A_hydrophila" = 2,

"A_jandaei" = 2,

"A_caviae" = 1,

"A_bivalvium" = 1

)

# Function to calculate percentage and 95% CI

calculate_ci <- function(count, total) {

test <- binom.test(count, total)

percentage <- (count / total) * 100

lower_ci <- test$conf.int[1] * 100

upper_ci <- test$conf.int[2] * 100

return(c(

Percentage = percentage,

CI_lower = lower_ci,

CI_upper = upper_ci

))

}

# Apply the function to all species

results <- t(sapply(species_counts, calculate_ci, total = total_isolates))

# Convert to data frame

results_df <- as.data.frame(results)

# Round values

results_df$Percentage <- round(results_df$Percentage, 2)

results_df$CI_lower <- round(results_df$CI_lower, 1)

results_df$CI_upper <- round(results_df$CI_upper, 1)

# Print results

print(results_df)
